# Correlation between mental health and pain-related impairment in patients with chronic cancer-related pain

**DOI:** 10.3389/fpsyg.2026.1793144

**Published:** 2026-05-22

**Authors:** Hannes Hofbauer, Felicitas Rapp, Kristin Kieselbach, Birgit Abberger, Ulrike M. Stamer, Stefan Wirz

**Affiliations:** 1 Pain Therapy Unit, Department of Anesthesiology and Intensive Care Medicine, University Hospital Ulm, Ulm, Germany; 2 Interdisciplinary Pain Center, Medical Center University Freiburg, Freiburg, Germany; 3 Department of Anesthesiology and Pain Medicine, Inselspital, Bern University Hospital, University of Bern, Bern, Switzerland; 4 Department for Anesthesiology, Intensive Medicine, Pain and Palliative Medicine, Cura Hospital/GFO-Clinics Bonn, Bad Honnef, Germany

**Keywords:** biopsychosocial pain model, chronic cancer-related pain, long-term survivors, mental health, patient-related outcome, quality of life

## Abstract

**Introduction:**

Cancer-related pain (CRP) has a high prevalence in cancer patients, with almost one-third of patients experiencing moderate to severe pain. Together with psychological problems, CRP is among the most common issues faced by cancer patients. According to the biopsychosocial pain model, pain is closely related to anxiety and depressive disorders, as well as poorer health-related quality of life (QoL) in cancer patients. Due to scarce data on changes in CRP levels and pain impairment in relation to mental disorders over time, this cross-sectional study investigated differences in mental health, QoL, and pain-related impairments depending on pain status and time intervals.

**Methods:**

A cross-sectional survey was conducted among cancer patients in an academic hospital pain clinic and members of support groups to assess CRP, pain-related impairment (Von Korff Severity Scale), depression, anxiety, stress (Depression, Anxiety, and Stress Scale [DASS]), and QoL (EORTC-QLQ-C30 Version 3). Participants were divided into six different groups by duration of cancer disease (<2 years, 2–5 years, and >5 years) and presence of CRP (pain/no pain).

**Results:**

A total of 256 datasets were evaluated. Patients with CRP (*n* = 119) showed poorer scores for depression (27.7% vs. 7.3%), anxiety (40.3% vs. 16.1%), stress (29.4% vs. 13.9%), and global QoL (53.8% vs. 16.1%) than patients without pain. The duration of cancer played a minor role in all comparisons between the groups. Female sex was associated with pain-related impairment, whereas a higher global QoL was associated with a slightly reduced odds ratio.

**Discussion and conclusions:**

Compared to patients without pain, CRP was associated with measurable differences in mental health at all time intervals in this cross-sectional survey, whereas the duration of cancer played a minor role. In the context of the biopsychosocial pain model, the potential negative impact of CRP on psychological well-being should be considered. Early identification of patients with CRP, followed by sufficient pain management and psycho-oncological/psychotherapeutic support for coping with pain in the biopsychosocial model, might improve long-term mental health and prevent pain chronification. In particular, the development of gender-sensitive interdisciplinary treatment approaches appears to have the potential to optimize care.

## 1 Introduction

The annual incidence of cancer is over 0.5 percent of the population in industrialized countries, and a further increase is expected in the next few decades (Quante et al., 2016; Rahib et al., 2021). Owing to improvements in diagnostics and oncological therapy, survival rates have also improved in recent years (Allemani et al., 2018). The number of long-term cancer survivors, defined as those whose initial cancer diagnosis was 5 years or more ago, is estimated to have risen in Europe from 9.9 million patients in 2010 to 14.5 million in 2020 (De Angelis et al., 2024). With an increasing number of cancer survivors, the focus has shifted to ensuring adequate care (Nekhlyudov et al., 2019). Among many other cancer-related impairments, persisting cancer-related pain (CRP) and mental disorders are two of the issues that most severely affect quality of life (QoL) (Götze et al., 2018). In cancer patients, depression and anxiety are the most common mental disorders (Kuhnt et al., 2016). According to a meta-analysis, over one-third of cancer survivors complain of chronic CRP, 22.8% of which is of moderate-to-severe intensity, resulting in personal suffering (Armoogum et al., 2023; Snijders et al., 2023).

Following the biopsychosocial pain model, a chronic pain disorder must be considered a disease in its own right (Treede et al., 2019). The biopsychosocial pain model provides a comprehensive framework for understanding chronic pain as a dynamic interaction between biological, psychological, and social factors (Novy and Aigner, 2014). A bidirectional influence between pain perception and depression has been described, leading to mutual reinforcement of symptom impairment (Kroenke et al., 2011; Bondesson et al., 2018). Failure to consider these relationships can lead to pain chronification. In addition to the known factors that contribute to chronic non-cancer pain, cancer-specific causes (e.g., release of pain-mediating inflammatory mediators) also play a role in the chronification of CRP and the extent of pain impairment. However, cancer-specific psychological factors, such as fear of recurrence or progression, are also important factors with a pronounced negative influence on pain perception (Broemer et al., 2021; Wirz et al., 2016). In line with the reciprocal relationship described in the biopsychosocial pain model, pain and psychological factors reinforce each other, resulting in negative effects on pain and pain-related impairments, as well as physical, psychological, and social functioning, leading to reduced QoL (Gnall et al., 2024). Mental disorders and poor QoL have potential negative impacts on cancer-specific mortality (Wang et al., 2020; Salabat et al., 2025). Sufficient pain control and adequate treatment of mental disorders, such as depression and anxiety, seem to be crucial in the care of cancer patients. It remains unclear whether, in addition to the pain state, the duration of cancer disease reveals differences in mental distress, such as depression, anxiety, stress, and QoL, or how pain-related impairment is associated with these mental health aspects, QoL, and duration of cancer illness. In this context, pain impairment is a more relevant parameter than pain intensity alone, as it encompasses the impact of pain and pain-related functional limitations on everyday life, recreational activities with family or friends, housework, and work.

To investigate these associations, in this cross-sectional survey, depending on the presence of CRP, patients were allocated to two groups (CRP-G *=* patient group with cancer-related pain; NP-G = patient group without pain) and then divided into three subgroups, each, depending on the time since initial cancer diagnosis, with the following intervals since initial cancer diagnosis: <2 years, 2–5 years, and >5 years.

We posed the following research questions:

- Is there a difference in mental distress and QoL depending on the duration of cancer (<2 years, 2–5 years, and >5 years) and presence of CRP (yes/no)?
- How is pain-related impairment associated with mental distress, QoL, and the duration of illness?

## 2 Methods

### 2.1 Implementation

We conducted a cross-sectional survey from May 2023 to October 2023. The survey comprised several validated questionnaires (see section Measures) and lasted approximately 15 min per participant. It was presented either as an online questionnaire or as a paper-and-pencil version. The online version was delivered via Unipark survey software (QuestBack GmbH).

Approval was granted by the Ethics Committee of the University of Ulm (Date: 10th May 2023/No.: 123/23), and the study was registered at the German Clinical Trials Register (ID DRKS00025282).

Participants were recruited from two different sources. The first group included patients who were treated for CRP in a pain clinic at a tertiary university hospital at least once between 2007 and 2023. The patients were requested in written form to participate in the study with the involvement of the cancer registry lists of the Comprehensive Cancer Center Ulm.

The second group of patients was recruited via e-mail. These were mainly individuals who attended support groups for cancer treatment. Overall, 23 support groups were contacted directly to spread the link to the survey to their group leaders. In addition, all 16 German regional cancer societies and a cancer patient information platform were used to spread the link to the survey.

The inclusion criteria for participation were a history of cancer in the past or at present, a minimum age of 18 years, sufficient knowledge of German, and cognitive capability to complete the questionnaire. Written informed consent was obtained from all the participants. The survey was conducted in accordance with the principles of the Declaration of Helsinki.

### 2.2 Measures

#### 2.2.1 Depression, anxiety, and stress scale (DASS)

The DASS measures depression, anxiety, and stress using three scales (Lovibond and Lovibond, 1995; Nilges and Essau, 2015). Higher scores indicate higher levels of depression, anxiety, and stress. Ten or more points on the depression and stress scales represent the cutoff for an increased probability of a depressive disorder or pronounced stress load. The same applies to six or more points on the anxiety scale, which represents the cutoff for an increased probability of an anxiety disorder.

#### 2.2.2 Quality of life questionnaire EORTC-QLQ-C30 version 3

The EORTC-QLQ-C30 Version 3 is a validated and standardized instrument for assessing the quality of life of patients with cancer (Aaronson et al., 1993). It comprises 30 items on five functional measures (physical, role, cognitive, emotional, and social); three symptom-oriented measures (fatigue, pain, and nausea/vomiting); and individual items to assess global quality of life. For global quality of life, a score of 50 was set as the cutoff score for poor quality of life in cancer patients (Diouf et al., 2015).

#### 2.2.3 Pain-related impairment

The Von Korff Severity Scale (Von Korff et al., 1992) measures pain-related impairment. The scores ranged from 1 to 4, where 1 represents low pain intensity and low pain-related impairment, 2 stands for high pain intensity and low pain-related impairment, 3 means high pain intensity and middle pain-related impairment, and 4 denotes high pain intensity and high pain-related impairment.

#### 2.2.4 Further questions

In addition, we assessed the presence of CRP as well as the duration of cancer disease [<2 years (representing initial oncological therapy and the primary processing phase), 2–5 years (representing the intermediate period), and > 5 years (representing the group of long-term cancer survivors)], age, and sex.

### 2.3 Statistical analyses

To describe the study population, descriptive statistics (mean [M], percentage [%], and standard deviation [SD]) were applied as distribution parameters, as well as boxplots. Histograms were analyzed to prove a normal distribution. As the variables were not normally distributed, global Kruskal–Wallis *U* tests were used to calculate group differences. For post-hoc analyses, pairwise Mann–Whitney *U* tests were used, and *p*-values were controlled using the Bonferroni–Holm method. No further multiple comparison correction was conducted owing to the explorative approach. To investigate the dependent variable pain-related impairment, a multivariable ordinal logistic regression with the independent variables including all three DASS subscales, global QoL, demographic variables, sex, and years since diagnosis was performed. All test results were considered explorative, and the level of significance was set to *α* = 0.05. Analyses were conducted using R version 4.5.1.

## 3 Results

### 3.1 Participants

#### 3.1.1 Hospital patient cohort

Overall, 691 patients provided written information regarding the study. A total of 101 patients expressed interest in participating in the study, and 82 patients completed and returned the questionnaire. The main reasons for non-participation (*n* = 590) were death of the patient, undeliverable postal letters, and no response. This corresponds to a participation rate of 11.86%.

#### 3.1.2 Support group cohort

The number of informed group members of self-help groups cannot be named because we do not know the exact number of addressed members of the individual groups. A total of 174 participants completed the questionnaire.

The two cohorts were collectively considered for further evaluation. They comprised 256 participants (169 females and 85 males). CRP was present in nearly half of the participants (CRP-G, 46.5% [*n* = 119]; NP-G, 53.5% [*n* = 137]). The participants were 60 years old on average (*M* = 60.4, SD = 13.1, range 26–89 years, *n* = 1 missing value). The most common cancers were gynecological (36.3%, *n* = 93), urological (16.4%, *n* = 42), and head and neck cancers (13.7%, *n* = 35). A total of 165 patients (60.9%) classified themselves as cancer-free (CRP-G 39.4%, *n* = 65; NP-G 60.6%, *n* = 100); 78 reported current recurrence, metastases, or progression (CRP-G 57.7%, *n* = 45; NP-G 42.3%, *n* = 33); and 10 were unable to provide any information about their actual cancer status (CRP-G 30.0%, *n* = 3; NP-G 70.0%, *n* = 10; *n* = 3 missing values). On average, 7.5 years had passed since the cancer diagnosis at the start of the survey (*M* = 7.6, SD = 7.1, *n* = 1 missing value) with 19.1% (*n* = 49; CRP-G 16.9%, *n* = 20; NP-G 21.2%, *n* = 29) suffering from cancer for less than 2 years, 27.3% (*n* = 70; CRP-G 32.2%, *n* = 38; NP-G 23.4%, *n* = 32) between 2 and 5 years, and 53.3% for more than 5 years (*n* = 136, CRP-G 50.8%, *n* = 60; NP-G 55.5%, *n* = 76; *n* = 1 missing value).

### 3.2 Differences in mental distress and QoL depending on cancer duration and CRP status

#### 3.2.1 Depression, anxiety, and stress scale

Mental distress was common, particularly among long-term cancer survivors, with higher scores for depression (14.7%), anxiety (27.2%), and stress load (19.1%). Regarding differences depending on pain status, 7.3% (*n* = 10) of participants without pain and 27.7% (*n* = 33) with pain reported increased scale values for depressive disorder. Elevated symptom scores above the cutoff for an anxiety disorder were observed in 16.1% (*n* = 22) of the participants without pain and 40.3% (*n* = 48) of the participants with pain. Moreover, 13.9% (*n* = 19) of the participants without pain and 29.4% (*n* = 35) with pain seemed to suffer from a pronounced stress load (Table 1).

**Table 1:** Descriptive variables of DASS scales, global QoL, and functioning scales.

Groups	CRP-G			NP-G		
	*M*	SD	*n*	*M*	SD	*n*
DASS scales						
Depression	14.6	5.3	118	11.0	4.3	137
Anxiety	12.9	4.3	116	10.3	3.4	135
Stress	16.1	4.8	114	12.0	4.5	135
EORTC-QLQ-C30 scales						
Global QoL	44.3	21.3	119	66.2	19.5	135
Physical functioning	60.7	20.2	116	79.7	19.1	137
Role functioning	39.9	24.1	119	68.2	24.6	133
Emotional functioning	42.68	22.8	115	65.2	23.0	134
Cognitive functioning	55.2	26.7	119	73.8	21.8	137
Social functioning	40.0	25.7	118	68.4	26.8	137

**Figure 1:**
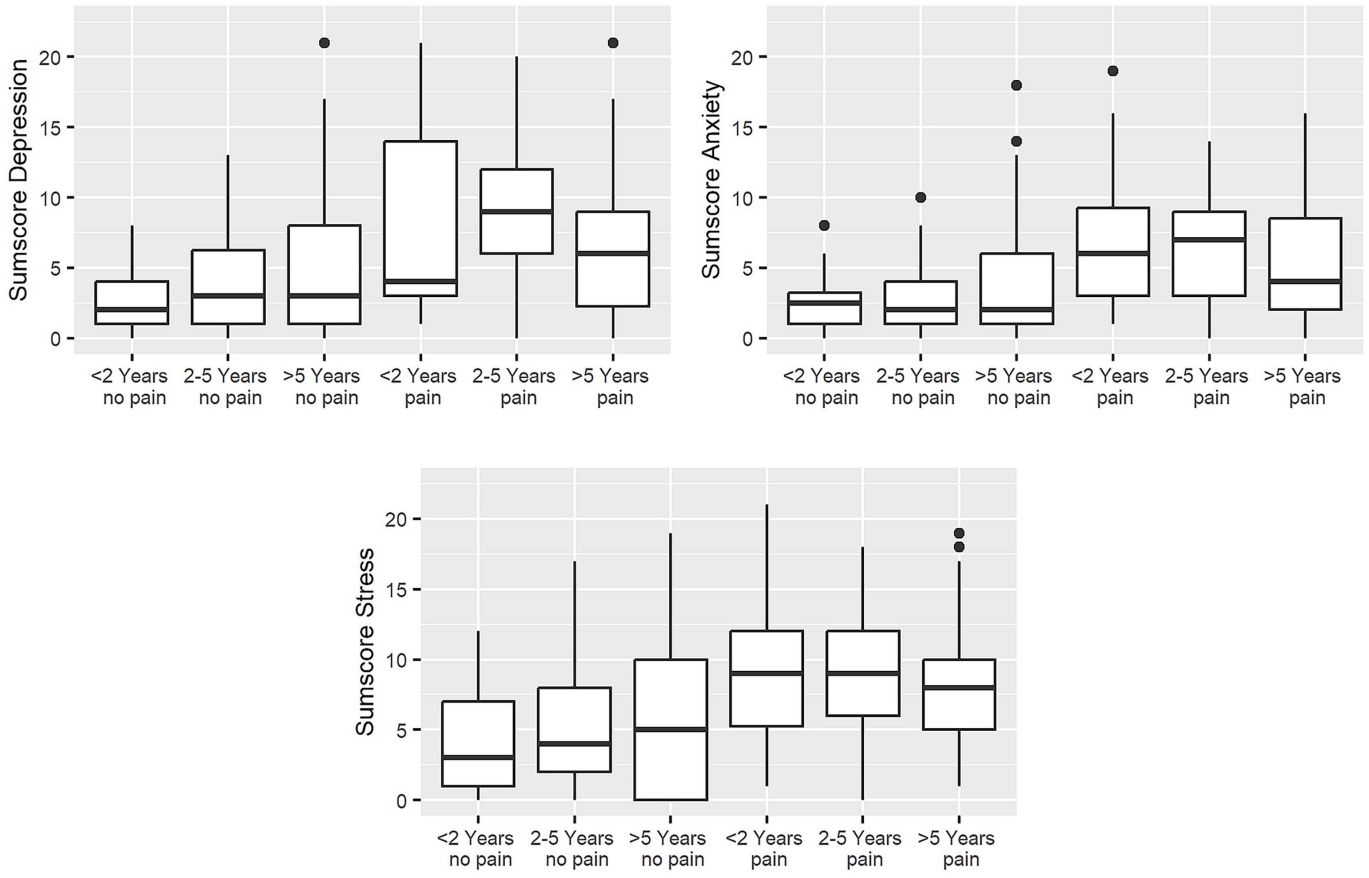
Boxplots of depression, anxiety, and stress sum scores depending on the time interval since initial cancer diagnosis and CRP status in 6 patient groups. The box depicts the median with the interquartile range (IQR), and whiskers from min to max; bullets represent outliers of 1.5 IQR. Depression: Kruskal–Wallis *χ*^2^ = 38.056, df = 5, *p*-value <0.001, *n* = 249, anxiety: Kruskal–Wallis *χ*^2^ = 31.02, df = 5, *p*-value <0.001, *n* = 248, stress: Kruskal–Wallis *χ*^2^ = 40.81, df = 5, *p*-value <0.001, *n* = 242.

In view of the duration of cancer disease, significant differences between the six duration groups (pain: <2 years, 2–5 years, and >5 years; no pain: <2 years, 2–5 years, and >5 years) were shown on the scales of depression, anxiety, and stress. Patients with pain revealed significantly higher scores at all examined time periods in the anxiety and stress scales than those without pain and in the depression scale at < 2 years, respectively. Overall, the variable cancer pain (yes/no) seems to have a greater influence, while the duration of cancer diseases led to no significant differences between the groups (see Table 2 and Figure 1).

**Table 2:** *P*-values of pairwise comparison—DASS.

Groups		NP-G			CRP-G	
		<2y	2–5y	>5y	<2y	2–5y
Depression scale						
NP-G	2–5y	0.21	–	–	–	–
	>5y	0.19	0.94	–	–	–
CRP-G	<2y	<0.05	0.05	0.05	–	–
	2–5y	<0.001	<0.001	<0.001	0.50	–
	>5y	<0.001	<0.01	<0.01	0.94	0.08
Anxiety scale						
NP-G	2–5y	0.86	–	–	–	–
	>5y	0.74	0.60	–	–	–
CRP-G	<2y	<0.01	<0.01	<0.01	–	–
	2–5y	<0.01	<0.01	<0.01	0.97	–
	>5y	<0.01	<0.01	<0.05	0.31	0.30
Stress scale						
NP-G	2–5y	0.44	–	–	–	–
	>5y	0.47	0.93	–	–	–
CRP-G	<2y	<0.01	<0.05	<0.05	–	–
	2-5y	<0.001	<0.001	<0.001	0.93	–
	>5y	<0.001	<0.001	<0.001	0.70	0.37

#### 3.2.2 Quality of life

More than half of the patients with pain (53.8%, *n* = 64) rated their QoL below the cutoff of 50; this was significantly less common in the group without pain (16.1%, *n* = 22). In addition to a higher QoL, participants without pain rated their functionality higher on all EORTC-QLQ-C30 scales than did participants with pain (Table 1).

**Figure 2:**
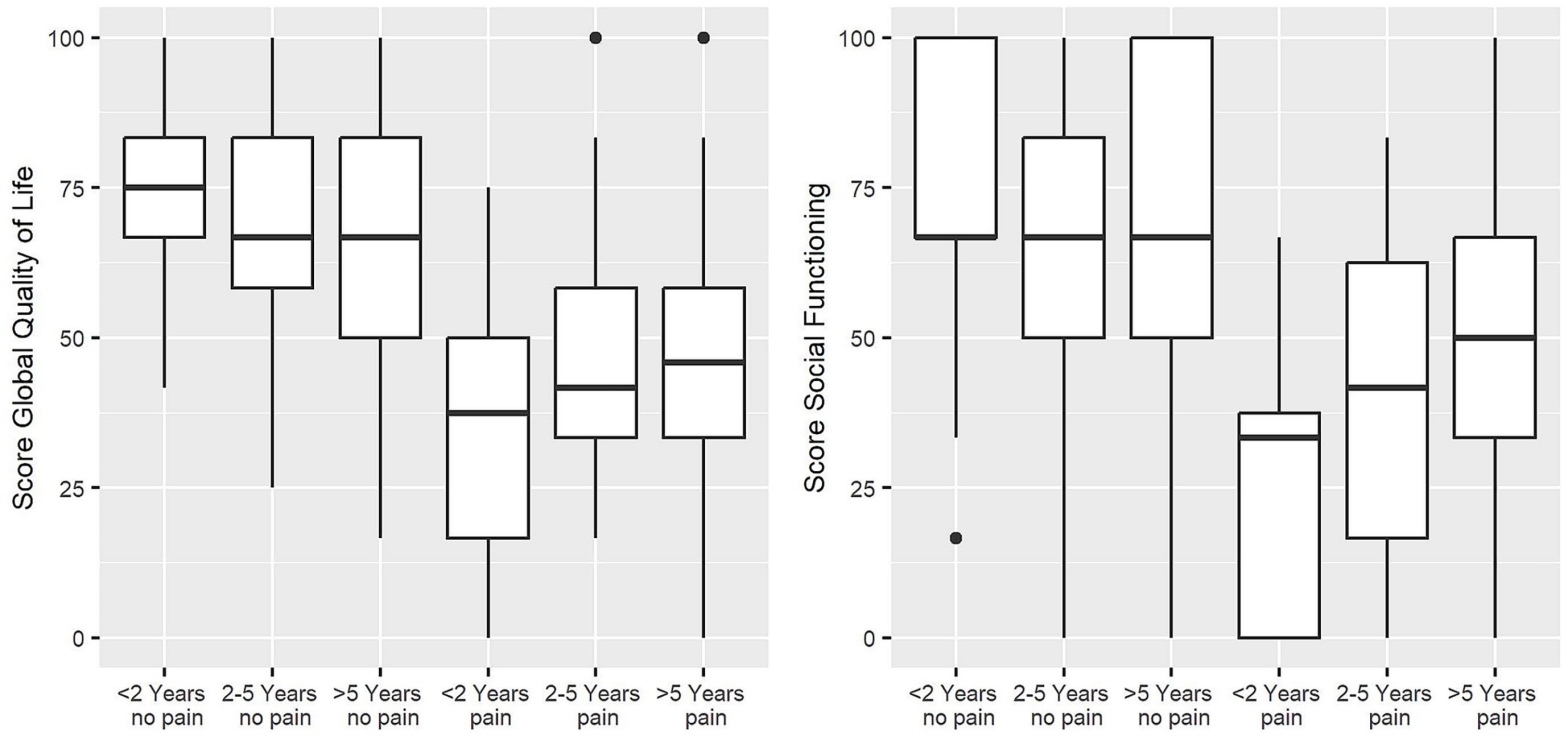
Boxplots of global QoL and social functioning sum scores depending on duration since initial cancer diagnosis and CRP status in 6 patient groups. The box depicts the interquartile range (IQR), and the whiskers show the min—max without outliers; bullets represent outliers of 1.5 IQR. Global QoL: Kruskal–Wallis *χ*^2^ = 64.64, df = 5, *p*-value <0.001, *n* = 253; social functioning: Kruskal–Wallis *χ*^2^ = 63.70, df = 5, *p*-value <0.001, *n* = 255; (not depicted: physical functioning: Kruskal–Wallis *χ*^2^ = 56.81, df = 5, *p*-value <0.001, *n* = 252; role functioning: Kruskal–Wallis *χ*^2^ = 65.63, df = 5, *p*-value <0.001, *n* = 251; emotional functioning: Kruskal–Wallis *χ*^2^ = 46.94, df = 5, *p*-value <0.001, *n* = 249; cognitive functioning: Kruskal–Wallis *χ*^2^ = 33.54, df = 5, *p*-value <0.001, *n* = 256).

There was also a difference in QoL across the six patient groups (duration since cancer diagnosis and pain [yes or no]). This group difference appears to be primarily explained by the presence of CRP. At all investigated time periods, participants with pain had significantly lower QoL and ability to function than those without pain. The duration of cancer also appears to play a subordinate role here (group comparisons over the investigated duration groups were almost insignificant; for more detail, see Table 3). Only global QoL and social functioning scales were influenced by the duration of cancer. On the global quality of life scale, the group of participants without pain showed that those with over 5 years since initial diagnosis (*M* = 63.2, SD = 20.0) had a significantly lower quality of life than those with less than 2 years of cancer history (*M* = 73.3, SD = 14.3). On the social functioning scale, there was a difference between the groups of participants with pain. Participants with less than 2 years since the initial cancer diagnosis reported significantly lower social functioning (*M* = 27.5, SD = 23.7) compared to those with a cancer history longer than 5 years (*M* = 45.2, SD = 24.3) (see Figure 2).

**Table 3:** *P*-values of pairwise comparison—EORTC-QLQ-C30 scales.

Groups		NP-G			CRP-G	
		<2y	2–5y	>5y	<2y	2–5y
Global QoL						
NP-G	2–5y	0.32	–	–	–	–
	>5y	<0.05	0.35	–	–	–
CRP-G	<2y	<0.001	<0.001	<0.001	–	–
	2–5y	<0.001	<0.001	<0.001	0.16	–
	>5y	<0.001	<0.001	<0.001	0.16	0.91
Physical functioning						
NP-G	2–5y	0.86	–	–	–	–
	>5y	0.37	0.28	–	–	–
CRP-G	<2y	<0.001	<0.001	<0.001	–	–
	2–5y	<0.001	<0.001	<0.001	0.26	–
	>5y	<0.001	<0.001	<0.001	0.24	0.98
Role functioning						
NP-G	2–5y	0.79	–	–	–	–
	>5y	0.43	0.79	–	–	–
CRP-G	<2y	<0.001	<0.001	<0.001	–	–
	2–5y	<0.001	<0.001	<0.001	0.38	–
	>5y	<0.001	<0.001	<0.001	0.38	0.90
Emotional functioning						
NP-G	2–5y	0.34	–	–	–	–
	>5y	0.57	0.68	–	–	–
CRP-G	<2y	<0.01	<0.01	<0.01	–	–
	2–5y	<0.001	<0.001	<0.001	0.68	–
	>5y	<0.001	<0.001	<0.001	0.47	0.68
Cognitive functioning						
NP-G	2–5y	0.53	–	–	–	–
	>5y	0.47	0.92	–	–	–
CRP-G	<2y	<0.001	<0.01	<0.01	–	–
	2–5y	<0.01	<0.05	<0.05	0.31	–
	>5y	<0.001	<0.01	<0.001	0.60	0.50
Social functioning						
NP-G	2–5y	0.27	–	–	–	–
	>5y	0.27	0.89	–	–	–
CRP-G	<2y	<0.001	<0.001	<0.001	–	–
	2–5y	<0.001	<0.001	<0.001	0.17	–
	>5y	<0.001	<0.001	<0.001	<0.05	0.31

#### 3.2.3 Determination of pain-related impairment

Nearly 80% of CRP-G experienced relevant pain-related impairment: 49.1% (*n* = 56) with high impairment, 30.7% (*n* = 35) with moderate impairment, 9.6% (*n* = 11) with high pain and low pain and low impairment, and 10.5% (*n* = 12) with low pain and low impairment, respectively (*n* = 114, 5 missing values).

Ordinal logistic regression shows that global quality of life and sex determine pain-related impairment. Higher QoL is slightly associated with a lower pain-related impairment. However, female sex doubles the probability of higher pain-related impairment (see Table 4 for more details).

**Table 4:** Variables associated with pain-related impairment (determined by the Von Korff Severity Scale) in CRP-G.

Variables	OR	CI (2.5–97.5%)	*p*-value
Depression	1.06	0.93–1.22	0.38
Anxiety	1.05	0.92–1.20	0.50
Stress	0.99	0.86–1.15	0.88
Global QoL	0.97	0.95–0.99	<0.05
Sex	2.62	1.06–6.54	<0.05
Cancer Duration	0.98	0.98–1.05	0.59

## 4 Discussion

The CRP is a general problem that often leads to a high psychosocial burden. To assess the influence of pain and pain impairment on mental health in subgroups divided into different time intervals after the initial cancer diagnosis, a cross-sectional survey was conducted. In summary, CRP led to detectable differences in mental health and QoL compared to patients without pain. In contrast, the duration of cancer appears to play a minor role in differences in mental distress, QoL, and pain-related impairment. Major pain-related impairments were associated with lower QoL and female sex.

The distribution of cancer entities among participants was comparable to that in Germany (Zentrum für Krebsregisterdaten and Robert-Koch-Institut, 2023). However, nearly two-thirds of the participants in our survey were female, whereas in the German cancer registry, more male than female patients with a new cancer diagnosis were recorded (Zentrum für Krebsregisterdaten and Robert-Koch-Institut, 2023). One explanation might be that women—besides having a slightly higher survival rate (Zentrum für Krebsregisterdaten and Robert-Koch-Institut, 2023)—tend to answer online surveys more likely than men (Smith, 2008). Almost half of the respondents suffered from CRP; over half of them were so-called long-term survivors with more than 5 years since the initial cancer diagnosis.

### 4.1 Mental distress

Mental disorders are common among cancer patients. In cancer patients, the lifetime prevalence rate was calculated to be 20.5% for depression and 24.1% for anxiety (Kuhnt et al., 2016). In our cohort, the rate of increased anxiety scores was higher than the reported lifetime prevalence. Focusing on long-term cancer survivors, the rates of anxiety and stress in our sample were higher than those described in a previous systematic review (Brandenbarg et al., 2019). However, individual studies have revealed a wide range of reported prevalence of mental disorders in cancer patients. This might be partly due to the different types of data collection (different questionnaires, interviews, etc.) and cancer entities investigated. Nevertheless, our complete sample showed a high mental health burden. One reason for this could be the differences in the presence and severity of pain. In our survey, participants with CRP were 2.2 to 3.7 times more likely to show increased scores over the cutoff for depression, anxiety, and pronounced stress than those without pain. However, it is important to recognize that pain and mental health disorders, such as depression, can reinforce each other in terms of the impact of their symptoms, and that potential causal links are even being discussed (Kroenke et al., 2011; Bondesson et al., 2018). Therefore, these factors cannot be viewed as entirely independent of one another.

In contrast, no cross-sectional differences in depression, anxiety, or stress load were observed across the respective duration groups with or without CRP. Longitudinal data on mental disorders over 5 years or longer in patients with cancer are limited; the majority of the studies are cross-sectional (Mehnert and Koch, 2008). Two longitudinal studies among breast cancer patients also revealed relatively constant scores in anxiety and depression over a time course of 5 years after the initial cancer diagnosis (Hopwood et al., 2010; Faye-Schjøll and Schou-Bredal, 2019). In summary, the existence of CRP seems to be closely connected to mental distress independent of the time course, which has rarely been considered in existing studies on the mental health of cancer patients.

Differences in QoL depend on the pain state. In the case of pain, respondents showed lower scores in global QoL and all functionality scales. The relevant impact of CRP on QoL is well described; this particularly applies to cancer survivors (Poço Gonçalves et al., 2021; Gao et al., 2023; Pérez et al., 2024), and the multidimensional impact of pain has recently been shown using a structural equation model (Hu et al., 2025). Again, there were minor differences between the duration groups in terms of the time since cancer diagnosis. Only global QoL in the NP-G was lower among long-term survivors compared to those with a short cancer history, while social functioning in the CRP-G was lower in the group <2 years compared to cancer survivors >5 years. The latter finding may be related to the high impact of newly diagnosed cancer and subsequent cancer therapy, which is often experienced as severely interfering. (Marzorati et al., 2025).

### 4.2 Pain-related impairment

Over 80% of CRP-G patients experienced a high degree of disability due to pain. Pain and pain-related impairment can affect nearly every aspect of daily life, including the ability to work (Armoogum et al., 2023). Chronic CRP is more common in women than in men (Gallaway et al., 2020; Karlson et al., 2020). Newer data have revealed genetic factors between migraine, multisite chronic pain, and breast cancer (Yang et al., 2025). However, data on whether female sex contributes to higher pain disability are controversial, and information in the case of CRP is scarce (Arnow et al., 2011; Hairi et al., 2013; Dahlhamer et al., 2018; Hellmann et al., 2025). In our survey, female sex was associated with an increased risk of pain-related impairment, whereas a higher global QoL was associated with a slightly reduced risk. In contrast, besides the duration of cancer disease, the factors depression, anxiety, or stress load revealed no influence on the pain disability that has been shown in other studies on non-cancer pain (Arnow et al., 2011; Von Korff et al., 2020).

### 4.3 Interpretation and potential for optimization

Mental disorders and low QoL are common problems in cancer patients, and many long-term cancer survivors experience lifelong suffering (Brandenbarg et al., 2019; Poço Gonçalves et al., 2021; Gao et al., 2023). Anxiety and depression appear to be linked to increased cancer mortality (Wang et al., 2020; Roche et al., 2023; Salabat et al., 2025). Furthermore, anxiety and fear of cancer recurrence seem to increase pain interference (Gnall et al., 2024). In contrast, the time course of cancer plays a subordinate role in mental disorders (Hopwood et al., 2010; Faye-Schjøll and Schou-Bredal, 2019). The presence of CRP revealed in our study had an extensive impact on mental health compared with those without pain in all duration groups. Even though our results can only be generalized partly due to their cross-sectional design and should not be taken as a causal inference, the impact on cancer survivors concerning chronic pain and mental health appears to be relevant. Therefore, prompt identification of patients with CRP followed by initiating sufficient pain therapy on a biopsychosocial basis at an early stage might help improve/stabilize mental health and QoL in cancer patients, preventing longstanding disability and pain chronification. From the view of healthcare professionals, raising awareness of CRP and the possible extensive pain modulation by psychosocial stress factors, such as depression or fear of recurrence/progression, seems to be fundamental. It is important to understand chronic CRP as a disease in its own right and not only as a symptom (Bennett et al., 2019; Treede et al., 2019). Furthermore, patients should be regularly screened for CRP and adequately treated, including low-threshold referrals to oncologists and, in severe cases and coexisting psychosocial stress factors to pain specialists. Repetitive education on pain, influencing factors, and treatment options will help patients search for sufficient help in pain therapy in a timely manner and should be an integral part of survivorship programs. In this context, the implementation of eHealth applications seems to be an important field to identify pain, pain impairment, and impaired mental health and to offer support to cancer patients at all stages. The majority of studies on eHealth have provided evidence of the possibility of alleviating CRP, but the observation periods were short (Hernandez Silva et al., 2019; van der Hout et al., 2021; Wu et al., 2024). However, eHealth applications seem to offer a great opportunity for long-term monitoring and support to improve the burden of chronic CRP substantially. Finally, in the case of chronic CRP (especially when accompanied by psychosocial stress factors), there is a necessity to develop comprehensive, specific pain therapy concepts, including interdisciplinary multimodal pain therapy (IMPT) programs that meet the special needs of cancer patients with pain (Hofbauer et al., 2025a, 2025b).

### 4.4 Limitations and strengths

Some limitations of this survey must be considered. The participation rate of the patients in the pain clinic was low, partly because a significant number of the patients had died or postal letters returned undeliverable. In addition, many of the patients who were contacted did not respond to the letters; it cannot be ruled out that this may have influenced participation, particularly among patients with CRP. As this group of participants had also been treated at the pain clinic at least once, a potential bias cannot be excluded (due to better pain treatment, more severely interfering pain, etc.). However, the participation rate of support groups cannot be named, as there is no information about how many members were contacted. Other potential influencing factors arise from the fact that the denominator in self-help groups cannot be clearly defined. Another selection and response bias might result from asking members of support groups, which are often attended to by more severely affected/concerned patients with higher levels of psychosocial burden, while coping with their situation might depend on the duration of their membership. Furthermore, our study had an overrepresentation of female participants, and the distribution of cancer entities might not be representative. The majority of participants in our study were over 50 years of age. Therefore, it was not possible to examine age-related differences, and the findings can only be applied to younger individuals to a limited extent. Despite being based on reasoned criteria, the time intervals for the duration groups were chosen arbitrarily. In particular, they do not reflect the highly heterogeneous course of individual patients. Questionnaires, even if validated, do not prove the presence of a specific mental disorder. As in the case of the DASS, when scores exceed a cutoff, they can only indicate an increased probability of depression, anxiety, or a stress disorder, which must be verified through clinical examination. Owing to the study design, this latter aspect could not be implemented. Therefore, its influence on the resulting prevalence of mental disorders cannot be ruled out. Pain etiology was not recorded because of the high risk of inaccurate data in the context of an online survey. Furthermore, due to the sample size, it was not possible to form subgroups according to cancer type, ongoing therapy, comorbidities, pain medication, etc. However, the focus was on CRP and pain impairment, not on cancer itself. Finally, owing to the cross-sectional design, conclusions on the differences between time intervals must be reflected carefully. The results can only be interpreted as associations and not as causal inferences between the presence of CRP and mental health disorders at different time intervals after cancer diagnosis.

In conclusion, several limitations restrict the generalizability of the results. Nevertheless, this study revealed the often disregarded or underrated relevance of CRP on mental health and QoL and can be used as a basis for further research.

## 5 Conclusion

The CRP and pain-related impairments are among the most common burdens experienced by cancer survivors and have a major impact on their mental health and QoL. Our data suggest that regardless of the time interval since cancer diagnosis, CRP is associated with significantly worse outcomes with regard to these parameters. In particular, when these problems occur in the early stages of cancer, comprehensive treatment of pain and mental disorders in an interdisciplinary setting based on the biopsychosocial pain model may help prevent pain chronification and other long-standing negative effects in long-term survivors. Since female cancer patients are at a particular risk for high pain impairment, gender-sensitive therapeutic approaches appear to be necessary.

## Data Availability

The datasets presented in this article are not readily available because of privacy reasons but are available from the corresponding author on reasonable request. Requests to access the datasets should be directed to hannes.hofbauer@uni-ulm.de.
